# Interactive effects of multiple stressors vary with consumer interactions, stressor dynamics and magnitude

**DOI:** 10.1111/ele.14013

**Published:** 2022-04-27

**Authors:** Mischa P. Turschwell, Sean R. Connolly, Ralf B. Schäfer, Frederik De Laender, Max D. Campbell, Chrystal Mantyka‐Pringle, Michelle C. Jackson, Mira Kattwinkel, Michael Sievers, Roman Ashauer, Isabelle M. Côté, Rod M. Connolly, Paul J. van den Brink, Christopher J. Brown

**Affiliations:** ^1^ Coastal and Marine Research Centre School of Environment and Science Australian Rivers Institute Griffith University Gold Coast Queensland Australia; ^2^ Naos Marine Laboratories Smithsonian Tropical Research Institute Balboa Ancón Republic of Panama; ^3^ 8001 College of Science and Engineering James Cook University Townsville Australia; ^4^ Quantitative Landscape Ecology iES—Institute for Environmental Sciences University Koblenz‐Landau Landau in der Pfalz Germany; ^5^ Research Unit of Environmental and Evolutionary Biology Namur Institute of Complex Systems and Institute of Life, Earth, and the Environment University of Namur Namur Belgium; ^6^ Wildlife Conservation Society Canada Whitehorse Yukon Territory Canada; ^7^ School of Environment and Sustainability University of Saskatchewan Saskatoon Saskatchewan Canada; ^8^ Department of Zoology University of Oxford Oxford UK; ^9^ 8748 Environment Department University of York York UK; ^10^ Syngenta Crop Protection AG Basel Switzerland; ^11^ Earth to Ocean Research Group Department of Biological Sciences Simon Fraser University Burnaby British Columbia Canada; ^12^ 396117 Aquatic Ecology and Water Quality Management Group Wageningen University Wageningen The Netherlands; ^13^ 396117 Wageningen Environmental Research Wageningen The Netherlands

**Keywords:** antagonism, consumer‐resource, seagrass, stressor interactions, synergy

## Abstract

Predicting the impacts of multiple stressors is important for informing ecosystem management but is impeded by a lack of a general framework for predicting whether stressors interact synergistically, additively or antagonistically. Here, we use process‐based models to study how interactions generalise across three levels of biological organisation (physiological, population and consumer‐resource) for a two‐stressor experiment on a seagrass model system. We found that the same underlying processes could result in synergistic, additive or antagonistic interactions, with interaction type depending on initial conditions, experiment duration, stressor dynamics and consumer presence. Our results help explain why meta‐analyses of multiple stressor experimental results have struggled to identify predictors of consistently non‐additive interactions in the natural environment. Experiments run over extended temporal scales, with treatments across gradients of stressor magnitude, are needed to identify the processes that underpin how stressors interact and provide useful predictions to management.

## Graphical abstract

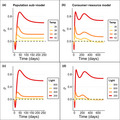

## INTRODUCTION

The global environment is under pressure from multiple stressors (Halpern et al., 2008; Jones et al., 2018). Many anthropogenic stressors are increasing, often at accelerating rates, so ecosystems will encounter novel conditions with increasing frequency (Halpern et al., 2019; Muhling et al., 2020). Traditional management generally focusses on reducing the impacts of single stressors. However, in the presence of multiple stressors, actions that mitigate single stressors can have drastically varying – and even detrimental – effects on ecological systems if potential interactions with other stressors are ignored (Brown et al., 2013). Therefore, accurate predictions of multiple stressor interactions are necessary for ecosystem management (Côté et al., 2016; Orr et al., 2020).

One way to predict stressor interactions is to identify generalities in types of interactions across ecosystems, stressors, levels of biological organisation, and timescales (Crain et al., 2008; Jackson et al., 2016; Tekin et al., 2020). A common approach is to classify stressor interactions as additive, antagonistic, or synergistic (Folt et al., 1999). Additive interactions occur when combined stressor effects equal the sum of each stressor considered in isolation; antagonistic interactions occur when the combined effects are less than the additive expectation; and synergies occur when interactions exceed the sum of the additive expectation (Folt et al., 1999; Piggott et al., 2015). Detecting synergistic interactions remains a focus in the literature, as anthropogenic stressors that interact synergistically will not only have negative effects on their own: they will also amplify negative impacts of other, co‐occurring environmental stressors (Côté et al., 2016; Darling & Côté, 2008).

Few consistent generalities have been found across different meta‐analyses (Côté et al., 2016; Crain et al., 2008; Jackson et al., 2016), as the identification of synergies and antagonisms is fraught with difficulties. First, interaction type is defined relative to a ‘null’ model (i.e. a model representing the expectation in the absence of stressor interactions), so the choice of null model (usually taken to be an expectation of additive effects) affects whether interactions are classified as synergies or antagonisms (Piggott et al., 2015; Schäfer & Piggott, 2018). Second, interaction classifications can differ depending on how the response variable is transformed prior to analysis. For example, an additive relationship on a log‐transformed scale is not additive, but multiplicative, on an arithmetic scale (Duncan & Kefford, 2021). Null hypotheses of interactions in linear model frameworks can thus be incorrect and misleading if the analytical decisions that can change the classification of stressor interactions are ignored (Griffen et al., 2016).

Interaction types can also change under different contexts. For example, interaction type can depend on population density, where higher population densities can mitigate the effects of multiple stressors on individual survival (Lange & Marshall, 2017). Changes in per capita interactions (both intra‐ and interspecific) or growth rates can counteract negative effects of environmental stressors when they reduce competition (Baert et al., 2018). Additionally, interactions can change with the presence of additional interacting stressors (Crain et al., 2008), stressor magnitude (Galic et al., 2018; Lange et al., 2018) or order of stressor exposure (Ashauer et al., 2017). The temporal dynamics of both responses and stressors create further challenges for quantifying stressor interactions (Jackson et al., 2021). The temporal scale at which the response is measured can affect the classification of stressor interactions (Garnier et al., 2017), while stressors themselves are not static in time and multiple stressors can impact systems through various discrete and/or continuous stressor events (Gunderson et al., 2016; Jackson et al., 2021). Progressing multiple stressor research requires moving beyond defining synergy or antagonism, and towards capturing the processes that mediate how stressors interact to influence different observed biological responses over multiple spatial and temporal scales (Simmons et al., 2021).

Most studies examining multiple stressors in recent decades have used linear models to evaluate interactions (e.g., ANOVA, linear mixed models). Non‐linear relationships between response and explanatory variables have been approximated using phenomenological approaches such as GAMs and polynomials. However a lack of mechanistic interpretability of model parameters when fitting such models means that generalities derived from such studies may yield misleading predictions of interactions (Duncan & Kefford, 2021; Orr et al., 2020). Models that capture the mechanisms that drive biological changes are required to predict ecosystem responses to multiple stressors (Schuwirth et al., 2019), particularly under novel conditions beyond the ranges of stressor values for which response data are available (Goussen et al., 2020). Without analysing stressor effects in a mechanistic or process‐oriented framework, the extent to which testing for synergy and antagonism can advance our understanding of multiple stressor interactions is likely to be limited (De Laender, 2018).

Process‐based models (PBMs) offer one way to predict ecological responses to multiple stressors (Haller‐Bull & Bode, 2019; Simmons et al., 2021; Tonkin et al., 2019). PBMs can be defined as ‘*models that characterize the changes in a system’s state as explicit functions of the events that drive those state changes*’ (Connolly et al., 2017). Stressor–response relationships are fundamental to PBMs, as they quantify the often non‐linear processes that drive change in biological responses and allow more informative predictions than methods such as linear statistical extrapolation because they explicitly model the mechanisms rather than fitting a phenomenological relationship between stressor and response (Griffen et al., 2016; Pirotta et al., 2022). Here, we integrate PBMs within a framework to classify interaction types and explore how two stressors interact to impact responses at physiological, population and consumer‐resource levels. Thus, we address a key research priority to examine patterns of stressor interactions at multiple levels of biological organisation (Orr et al., 2020) and ecological scales (Simmons et al., 2021). We demonstrate our approach using seagrass meadows challenged by increased temperature and light limitation – caused by climate warming and poor water quality associated with land‐use change, respectively.

## MATERIALS AND METHODS

### Model study system

Seagrass meadows are one of the world’s most important coastal habitats and a model system for multiple stressor experimental studies (Stockbridge et al., 2020). They provide ecosystem services including contributions to fisheries (Unsworth et al., 2019), carbon sequestration (Macreadie et al., 2014) and coastal protection (Koch et al., 2009). Seagrass meadows are threatened by multiple stressors such as ocean warming and poor water quality (Dunic et al., 2021; Thomson et al., 2015; Turschwell et al., 2021). The cumulative effects of such stressors will continue affecting seagrass habitats, as oceans continue to warm (Chefaoui et al., 2018) and land‐based activities degrade coastal ecosystems (Saunders et al., 2017). We calibrated a PBM using parameter estimates from published empirical studies and single‐stressor models calibrated to empirical data for the tropical seagrass genus *Halodule*, which is generally classified as having colonising or opportunistic life‐history traits (Kilminster et al., 2015). Our models characterize the expected outcomes of experiments where treatments are being compared under different stressors levels (hence we refer to modelled stressor responses as ‘model runs’). Structurally similar models could easily be used to explore multiple‐stressor effects on other marine, freshwater or terrestrial primary producers.

### Models

#### *Physiological sub*‐*model*

At a physiological level, temperature and light affect gross photosynthesis, whereas only temperature affects respiration (Adams, Koh, et al., 2020). Photosynthesis rate, $P\left(I,T\right)$, was modelled as a non‐linear function of irradiance, *I*, and temperature, *T*:

(1) $$P\left(I,T\right)=P_{\text{max}}\left(I,T\right)\left(1‐\frac{B}{B_{\text{max}}}\right)$$

where B is the above ground biomass in a monotypic stand, proportional to $B_{\text{max}}$, which is the biomass at which gross production is zero. $P_{\text{max}}$ is the maximum specific production, at temperature *T* (°C) and irradiance *I* based on the Jassby–Platt parameterisation (Jassby & Platt, 1976):

(2) $$P_{\text{max}}\left(I,T\right)=P_{T\_\text{max}}\left(\frac{T_{\text{max}}‐T}{T_{\text{max}}‐T_{\text{opt}}}\right){\left(\frac{T}{T_{\text{opt}}}\right)}^{T_{\text{opt}}/\left(T_{\text{max}}‐T_{\text{opt}}\right)}\text{tanh}\left(\frac{I}{I_{k}}\right),$$

where $I_{k}$ is the saturation irradiance, $P_{T\text{max}}$ is the maximum gross production at temperature *T* derived from the Yan and Hunt model (Adams et al., 2017; Yan & Hunt, 1999), $T_{\text{max}}$ is the maximum temperature at which photosynthesis can occur and $T_{\text{opt}}$ is the optimal temperature for photosynthesis.

The photosynthesis model assumed no photo‐inhibition at high irradiance (Fourqurean & Zieman, 1991) and included a logistic function to capture self‐shading of the plant canopy, which limits production at high shoot densities (Burd & Dunton, 2001). Oxygen (O_2_) consumption rates were converted to carbon (C) fixation rates by assuming that the amount of carbon fixed/released during photosynthesis and respiration was equal to the amount of O_2_ evolved/fixed, respectively (Adams et al., 2017; Roberts & Moriarty, 1987). Leaf respiration, $R\left(T\right)$, was modelled as a non‐linear function of temperature *T*:

(3) $$R\left(T\right)=R_{\text{max}}\left(\frac{R_{T\_\text{max}}‐T}{R_{T\_\text{max}}‐R_{T\_\text{opt}}}\right){\left(\frac{T}{R_{T\_\text{opt}}}\right)}^{R_{T\_\text{opt}}/\left(R_{T\_\text{max}}‐R_{T\_\text{opt}}\right)},$$

where $R_{\text{max}}$ is the maximum respiration at temperature *T*, also derived from the Yan and Hunt model, $R_{T\_\text{max}}$ is the maximum temperature at which respiration can occur, and $R_{T\_\text{opt}}$ is the optimal temperature for respiration. Finally, we defined net production per *B* per day (response variable for the physiological sub‐model), $N_{P}$, as:

(4) $$N_{P}=P\left(I,T\right)‐R\left(T\right).$$

#### *Population sub*‐*model*

For the population sub‐model, we converted grams of carbon to grams of seagrass tissue, assuming that carbon accounts for approximately 33% of plant biomass (Hansen et al., 2000). We modelled change in biomass (g dry weight m^–2^) as a dynamic state variable, *B*(*t*), following:

(5) $$\frac{dB\left(t\right)}{\mathrm{dt}}=P\left(I,T\right)B\left(t\right)‐R\left(T\right)B\left(t\right)‐mB\left(t\right),$$

where m is mortality, defined by the rate of seagrass leaf loss. The analytical solution to this differential equation gives biomass as an explicit function of time:

(6) $$B\left(t\right)=\frac{\beta F\exp\left(\beta t\right)}{1‐\alpha F\exp\left(\beta t\right)},$$

where tis the time in days, the initial biomass ($B_{0}$) is used to compute $F=\frac{B_{0}}{\alpha B_{0}+\beta }$, and $\alpha =\frac{‐P_{\text{max}}\left(I,T\right)}{B_{\text{max}}}$ and $\beta =P_{\text{max}}\left(I,T\right)‐R\left(T\right)‐m$.

#### *Consumer*‐*resource model*

To model dynamic interactions between the primary producer and a consumer we added a term representing biomass consumption by the consumer (*X(t)*):

(7) $$\begin{array}{c} \frac{dB\left(t\right)}{\mathrm{dt}}=P\left(I,T\right)B\left(t\right)‐R\left(T\right)B\left(t\right)‐mB\left(t\right)\\ \;‐aS\left(T\right)B\left(t\right)X\left(t\right). \end{array}$$

Here, *a* is the consumer attack rate, $S\left(T\right)$ is the temperature‐dependent scaling equation for the consumer attack rate and mortality (discussed in López‐Urrutia, 2008), based on the metabolic theory of ecology (Brown et al., 2004):

(8) $$S\left(T\right)=s_{0}\exp\left(\frac{‐E}{k\left(T+273.15\right)}\right),$$

where $s_{0}$ is the body size dependent normalization factor of the metabolic rate, *E* is the activation energy of heterotrophs and *k* is Boltzmann’s constant (Table 1). This equation is assumed to be the same for temperature scaling of the attack rate and mortality based on previous studies (O’Connor et al., 2011).

**TABLE 1:** Parameters used in the model. Values in brackets indicate range of values tested in sensitivity analyses

Parameter	Definition	Value	Species	Units	Reference
$T_{\text{max}}$	Maximum temperature for photosynthesis	44.5 (35–50)	*Halodule uninervis*	°C	(Adams et al., 2017)
$T_{\text{opt}}$	Optimal temperature for photosynthesis	34.9 (20–40)	*H. uninervis*	°C	(Adams et al., 2017)
$R_{T\text{max}}$	Maximum respiration temperature	45.6 (40–50)	*H. uninervis*	°C	(Adams et al., 2017; Collier et al., 2017)
$R_{T\text{opt}}$	Optimal respiration temperature	39.1 (25–45)	*H. uninervis*	°C	(Adams et al., 2017; Collier et al., 2017)
$R_{\text{max}}$	Maximum gross respiration	0.08	*H. uninervis*	g C dry weight d^−1^	(Adams et al., 2017; Collier et al., 2017)
$P_{T\text{max}}$	Maximum gross production at temperature *T*	0.34	*H. uninervis*	g C dry weight d^−1^	(Adams et al., 2017)
$I_{k}$	Saturation irradiance	319	*Halodule wrightii*	μmol m^−2^ s^−1^	(Burd & Dunton, 2001)
$B_{\text{max}}$	Carrying capacity of the canopy	667	*H. wrightii*	g dry wt m^–2^	(Burd & Dunton, 2001)
*m*	Mortality (leaf loss rate)	0.004	*H. wrightii*	Above ground biomass d^−1^	(Burd & Dunton, 2001)
*a*	Attack rate	0.001 (0.00001–0.1)		m^2^ d^−1^	*parameterized for model stability
*c*	Assimilation efficiency	0.15		g ^−1^ dry wt	(Adams, Sisson, et al., 2020; Law et al., 2016)
*v*	Consumer mortality rate	0.05		d^−1^	*parameterized for model stability
$s_{0}$	Body‐size‐dependent normalization factor of the metabolic rate	4.306e10		dimensionless	
*k*	Boltzmann constant	8.617e−5		eV K^−1^	
*E*	Activation energy of heterotroph	0.65		eV	(López‐Urrutia, 2008)

We modelled the change in consumer density over time, *X*(*t*), as:

(9) $$\frac{dX\left(t\right)}{\mathrm{dt}}=acS\left(T\right)X\left(t\right)B\left(t\right)‐vS\left(T\right)X\left(t\right),$$

where *X* is the consumer density (m^−2^), c is the consumer assimilation efficiency, and v is the consumer’s mortality rate. Assimilation efficiency was set at 15% (Table 1).

### Model runs

**FIGURE 1:**
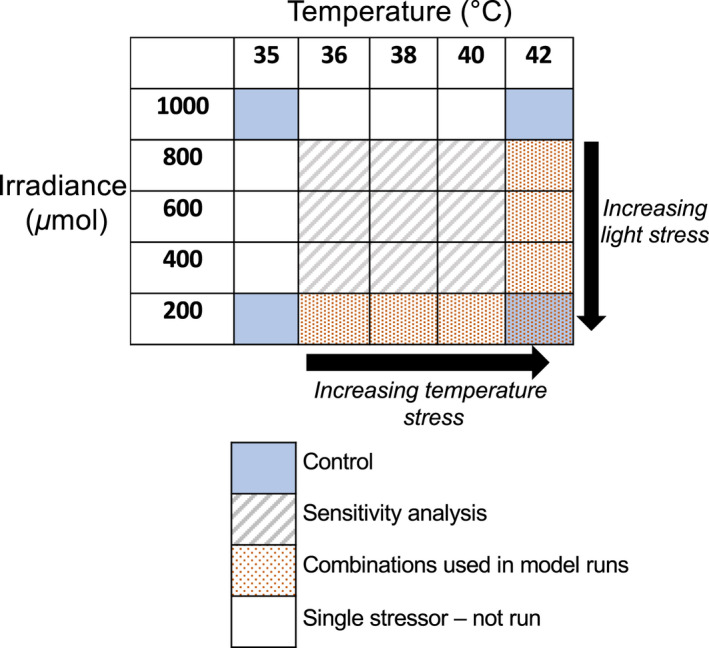
Illustration of stressor levels used for model runs. Blue boxes indicate model runs under optimal (control) conditions ($T_{\text{opt}}$ and $R_{\text{opt}}$ in Table 1 – top left box), under maximum light stress but at optimal temperature (bottom left), at maximum temperature stress but optimal light (top right), and under both maximum temperature and light stress to represent multiple co‐occurring stressors (bottom right). Legend indicates combinations of temperature and light stressor intensities used in model runs to assess multiple stressor interactions and combinations tested in sensitivity analyses (results shown in the supplementary material)

We solved the models (either analytically for physiological or population models, or numerically for the consumer‐resource model) for a certain parameter set, to obtain dynamics of state variables, which were then used to calculate stressor interaction statistics (next section). We ran four scenarios (Figure 1 – blue panels) to explore how seagrass biomass changes through time under different combinations of stressor magnitude. We assumed that stressor magnitude remains constant but acknowledge this is rarely the case in nature (Jackson et al., 2021). Seagrass biomass was modelled under (i) optimal (control) conditions (Figure 1 – top left blue box), (ii) maximum light stress (i.e. lowest light available for photosynthesis) but optimal temperature (Figure 1 – bottom left blue box), (iii) maximum temperature stress but optimal light (Figure 1 – top right blue box) and (iv) both maximum temperature and light stress to represent multiple co‐occurring stressors (Figure 1 – bottom right blue box). We modelled responses to a suite of multiple stressor combinations (Figure 1 – orange‐dotted boxes) to calculate stressor interactions and demonstrate how responses at different levels of organisation can vary along stressor gradients that deviate from optimal conditions. Starting seagrass biomass was set at 10% of $B_{\text{max}}$ for all model runs.

### Classifying multi‐stressor interactions

We defined stress as any deviation from ‘optimal’ conditions that affect photosynthesis and respiration. We recognise that different stressor interaction statistics are more appropriate for capturing true interactions in different scenarios (*sensu* Folt et al., 1999). Thus, we use two different metrics for capturing two responses of a different nature. First, let $Y_{A},Y_{B},Y_{C}$ and $Y_{\mathrm{AB}}$ be the response variable of the treatment with stressor A only, stressor B only, no stressors (control – C), and both stressors (A and B), respectively. To assess the stressor interaction type, we can define the interaction statistic ρ:

(10) $$\text{ρ}=‐ln\left(\frac{Y_{\mathrm{AB}}/Y_{C}}{Y_{A}Y_{B}/Y_{C}^{2}}\right).$$

This metric, ρ assesses whether the rate of change of the response in the presence of both stressors is greater than the multiplication of the rate of change due to individual stressor effects. We classify the interaction as synergistic when ρ is positive, additive when ρ is zero and antagonistic when ρ is negative. Using this statistic is natural when it is expected that stressors will affect a response measured as a rate, which is the case for the population and consumer models because biomass is multiplied by a growth rate which depends on the stressor levels (i.e. the stressors have a multiplicative effect on the response). However, for the physiological sub‐model, using ρ would be an unnatural choice because the response variable $N_{P}$ is clearly not affected by either stressor multiplicatively (this can be seen by inspection from Equation (4), as there is no $N_{P}$ on the right‐hand side). Thus, we define a different interaction statistic, δ, to assess the physiological sub‐model, where (with $N_{P}$ as the response variable *Y*):

(11) $$\delta =‐\left(\left(\left(Y_{C}‐Y_{A}\right)+\left(Y_{C}‐Y_{B}\right)\right)‐\left(Y_{C}‐Y_{\mathrm{AB}}\right)\right).$$

Similarly, we classify the interaction as synergistic when δ is positive, additive when δ is zero, and antagonistic when δ is negative.

### Sensitivity analysis

We tested the sensitivity of our models to a range of values of the parameters $T_{\text{max}}$, $T_{\text{opt}}$, $R_{T\text{max}}$, $R_{T\text{opt}}$ and *a* (varied one at a time), to examine changes in interaction type and strength. We also implemented three alternative model forms to test how functional forms of key processes at each level of organization affected the stressor interaction strength and type. For the population model, we altered the form of density‐dependence by implementing a Gompertz function as an alternative to the logistic. We also re‐parameterised our physiological model so that temperature and light affect biomass indirectly (parameter $P_{\text{max}}$ affecting equilibrium biomass). For the consumer‐resource model, we implemented a Holling Type II functional response for the consumer feeding on seagrass.

## RESULTS

We found that the same processes can underpin additive, synergistic, and antagonistic stressor interactions. The strength and type of interaction depended mostly on when the interaction statistic was quantified (relative to time needed to reach equilibrium), the magnitude of stressor effects and the presence of a consumer. We discuss these findings in more detail below for each of our three models.

### *Physiological sub*‐*model*

**FIGURE 2:**
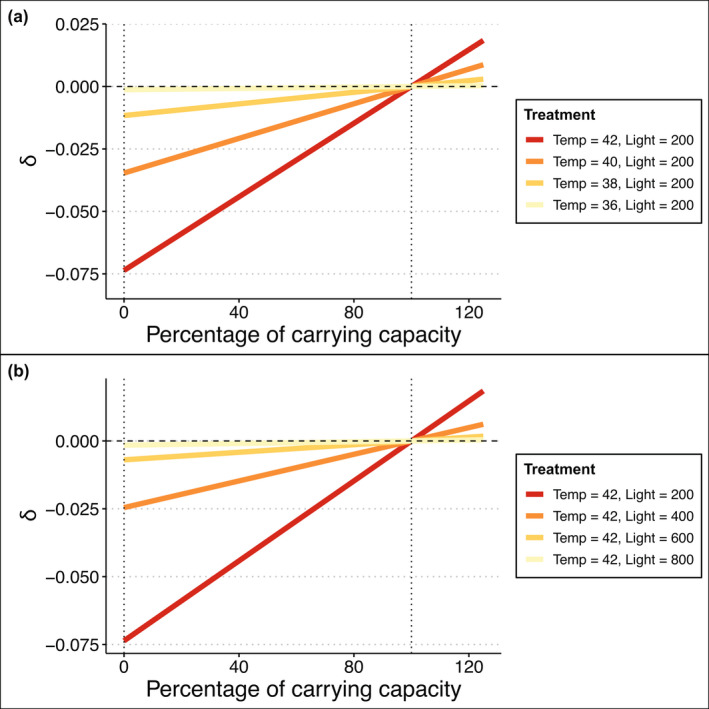
The interaction metric used to assess the physiological sub‐model, δ, as a function of proportion of carrying capacity achieved through the net production of seagrass under multiple stressor scenarios for (a) fixed light stress (200 *μ*mol) but varied temperature stressor magnitude and (b) fixed temperature stress (42°C) but varied light stressor magnitude. The interaction metric δ is linearly related to biomass because it is the sum of several other quantities that are linearly related to biomass. Positive values of δ indicate synergistic interactions, while negative values indicate antagonistic responses of seagrass net production. Dashed line where δ= 0 denotes where interactions are additive. Dotted vertical lines are at 0% and 100% of carrying capacity. The red line represents the highest temperature and light stress combination, whereas the lightest yellow line represents the lowest multiple stressor combination (orange textured boxes in Figure 1)

The interaction metric δis linearly related to biomass because it is the sum of several other quantities that are linearly related to biomass. For fixed temperature and light levels, $N_{P}$is linearly related to biomass with a positive slope (Figure 2). Slope steepness and the $N_{P}=0$intercept depend on temperature and light, where the intercept at $N_{P}=0$occurs at lower biomass levels when stressor intensity is greater. Additionally, when biomass is at carrying capacity, net production is zero, and $N_{P}=‐R\left(T\right)$. This is a finding that holds across all temperatures and is independent of light level.

For any combination of light and temperature, interaction type was antagonistic for all biomass values below carrying capacity; however, interaction type reversed such that all interactions became synergistic for all stressor treatments when biomass exceeded carrying capacity (Figure 2). The stressor interaction type was approximately additive at the carrying capacity because $N_{P}=‐R\left(T\right)$. Generally, stressor interactions were antagonistic when temperature deviated from the thermal optimum (assuming photosynthesis without shading exceeds respiration). However, synergistic interactions could occur when applying temperature ‘stressors’ that shift toward thermal optima or when respiration exceeded photosynthesis (without shading) – which represents an unviable population. For stressors that reduced net growth, interactions were always antagonistic.

### *Population sub*‐*model*

**FIGURE 3:**
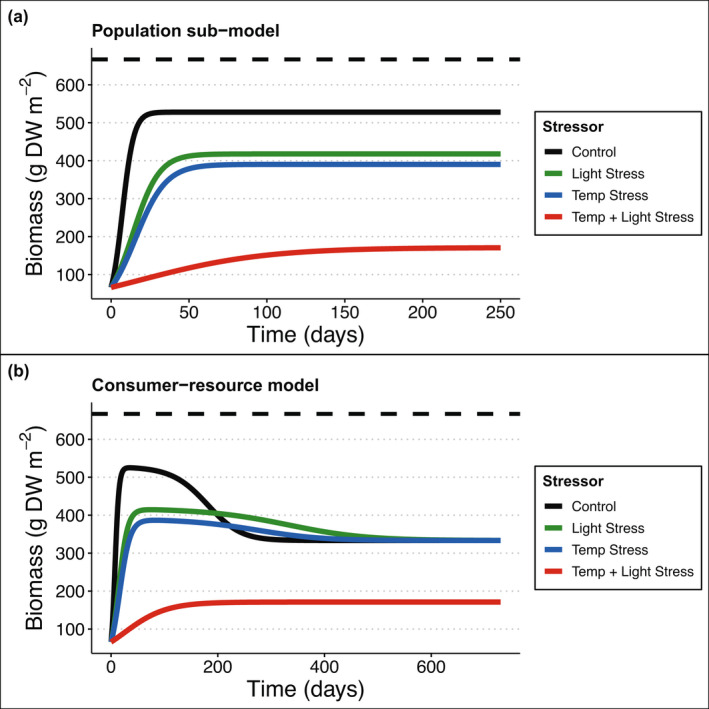
Temperature and light interact non‐linearly to affect seagrass biomass in (a) the population sub‐model, and (b) the consumer‐resource model. Dashed line in panels represent $B_{\text{max}}$ of 667 g dry weight m^–2^. Initial biomass was fixed at 10% of $B_{\text{max}}$. Scenarios are control conditions (black), maximum light stress but control temperature (blue), maximum temperature stress but optimal (control) light (green) and both maximum temperature and maximum light stress (red). Note different durations of model runs on X axis

Under optimal (control) conditions, biomass rapidly increased while population growth was in the exponential phase of the logistic curve, and then slowed because of shading before reaching equilibrium (when net production is zero) at a biomass of approximately 80% of $B_{\text{max}}$, which took ~25 days (Figure 3a). Under both single stressor scenarios (i.e. only light stress or only temperature stress – blue boxes in Figure 1), population growth was slower compared with control conditions, the system took more than twice as long to reach equilibrium, and biomass stabilised at approximately 60% of $B_{\text{max}}$ (Figure 3a). Finally, under maximal stress from two co‐occurring stressors, population growth was drastically slower compared with optimal conditions, the system took more than 10 times longer to reach equilibrium, and biomass stabilised at approximately 25% of $B_{\text{max}}$ (Figure 3a).

**FIGURE 4:**
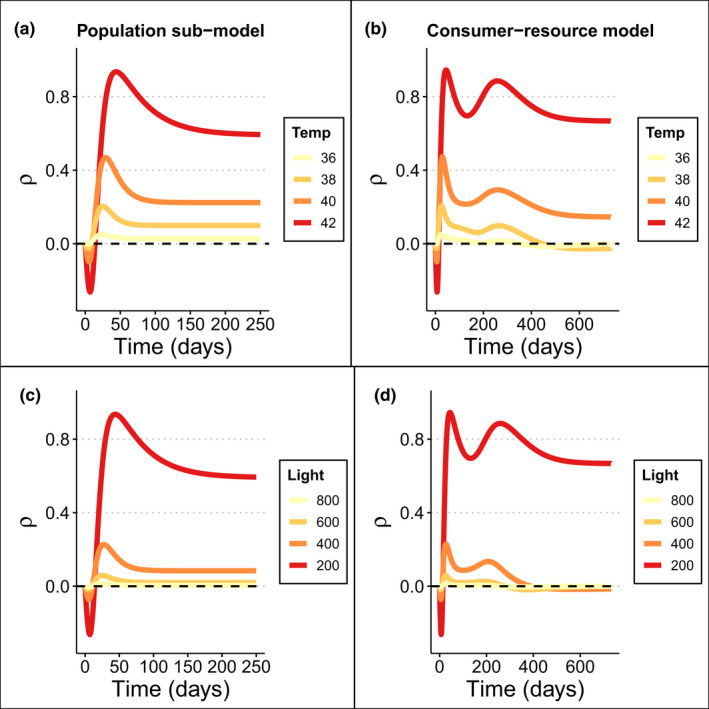
Stressor interactions as a function of time for the seagrass population sub‐model (a, c) and consumer‐seagrass model (c, d) under multiple stressor scenarios. Scenarios are fixed light stress (200 *μ*mol) but varied temperature stressor magnitude (a, b), and fixed temperature stress (42°C) but varied light stressor magnitude (c, d). ρ is the interaction metric, where positive values indicate synergistic interactions between stressors, and negative values indicate antagonistic interactions. Dashed line at zero denotes where interactions are additive

Using the multiplicative interaction metric, ρ, we found that for all temperature (Figure 4a) and light stressor scenarios (Figure 4c), the same stressors could interact antagonistically, additively, or synergistically depending on stressor magnitude, and on when the interaction statistic was quantified, relative to the timescale over which the system approached its asymptotic behaviour (e.g. reached equilibrium). Over short timescales (<25 days) – during the exponential phase of population growth – stressor interactions were generally antagonistic, but over longer times‐scales (>25 days), interactions were synergistic (Figure 4a). Additionally, the stressor interaction tended to increase (i.e. trend towards synergy) as stressor magnitude increased (Figure 4a, c). Stressor interaction strength also varied through time, until the system reached equilibrium. At equilibrium, there were no antagonistic interactions, and the only approximate additive interaction occurred under high temperature stress and low light stress (yellow line in Figure 4c).

Across all scenarios, stressor interactions depended on the timescale at which the interaction was measured due to differential growth rates and shading effects under each stressor combination. For example, growth initially dominated the change in population biomass, as all treatments were in the exponential phase of logistic growth (Figure 3a). Growth rate was highest in the control treatment, leading to faster growth and therefore proportionally higher biomass compared with stressed treatments. This led to antagonistic stressor interactions because all stressed treatments were at low biomass relative to the control (Figure 4a, c). When shading began to reduce growth rate in the control treatment, the single and combined stressor treatments remained in the exponential growth phase (Figure 3a), leading to increased synergistic stressor interactions because the relative difference between the biomass in the single stressor treatments versus the control was much lower than in the multiple stressor treatment versus the control (Figure 4a, c). In the next growth phase, the change in biomass in the control and the single stressor treatments was limited by self‐shading, while the multiple stressor treatment remained in the exponential phase of growth (Figure 3a). Thus, interactions became less synergistic over time until all treatments reached equilibrium, and the long‐term stressor interaction level (i.e. all synergistic) was realised (Figure 4a, c).

### *Consumer*‐*resource model*

The consumer‐resource model behaved similarly to the population model. Under control growth conditions, the consumer‐resource model experienced rapid initial growth, before declining as the consumers reduced seagrass biomass. The consumer and resource reached equilibrium at ~300 days, when seagrass biomass was approximately 50% of $B_{\text{max}}$ (Figure 3b – dark blue line). Therefore, seagrass biomass had a slower, more oscillatory approach to equilibrium in the presence than in the absence (c.f. population sub‐model, Figure 3a) of a consumer. The presence of a single stressor reduced the impacts of the consumer on seagrass biomass (i.e. for a period of time seagrass biomass was higher in the presence of a stressor compared with control conditions). When either light or temperature stress were acting alone, seagrass and consumer coexisted and seagrass reached the same biomass equilibrium as under control conditions, albeit more slowly (~150 days longer: Figure 3b). The presence of multiple stressors negated any compensatory effects, and seagrass growth was further slowed. In this case seagrass biomass was not sufficient to support a consumer, so the consumer became extinct, and the system approached the single‐population equilibrium (~25% of $B_{\text{max}}$; Figure 3b).

**FIGURE 5:**
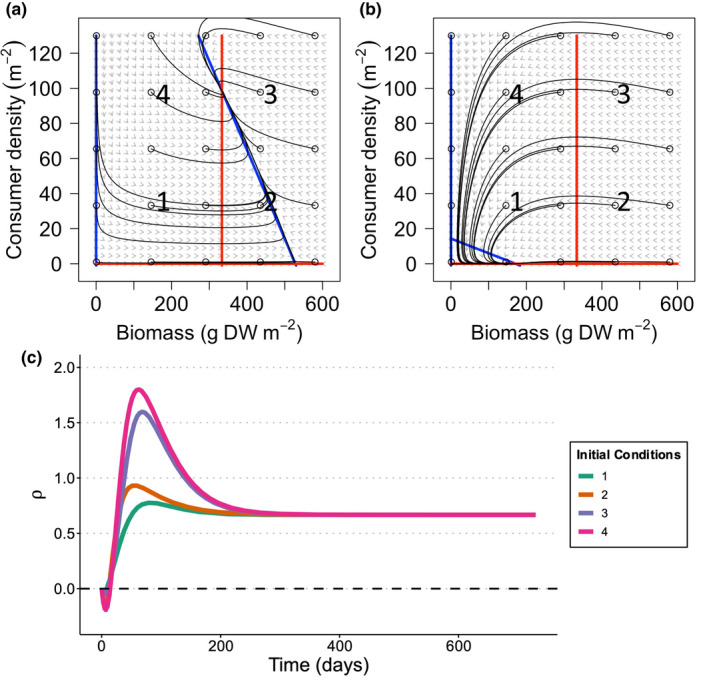
Phase plots under (a) no stress (= optimal, control conditions) and (b) multiple stressors in the consumer‐resource model. Grey arrows indicate the direction of movement and relative magnitude of a particle state change. Blue lines and red lines represent the seagrass‐nullclines and consumer‐nullclines, respectively, and identify states where seagrass and the consumer are at equilibrium. Black lines represent the trajectories of the system, when starting from different initial conditions. Circled numbers (1–4) indicate different initial model conditions and panel c shows behaviour of ρ for each set of initial conditions in panel b

Using the multiplicative interaction metric, ρ, for all temperature (Figure 4b) and light stressor scenarios (Figure 4d), the same stressors could interact antagonistically, additively, or synergistically depending on stressor magnitude, consumer effects, and when the response was measured relative to the system’s long‐run state. As per the population model, over short timescales (<25 days), during the exponential growth phase, stressor interactions were antagonistic. After this phase, a similar pattern to the population model was observed, with the same interpretation based on growth and shading processes. However, there was an extra phase where the biomass of seagrass in the control and single stressor treatments moved above the equilibrium biomass prior to being drawn down by the consumer (Figures 4b, d and 5a). This led to an extra synergistic peak at ~250 days, where the biomass in the single stressor treatments exceeded control biomass due to a higher consumer population in the control treatment. Unlike the population sub‐model, over longer timescales (>25 days), multiple stressors could change between antagonistic, additive or synergistic effects in the presence of a consumer (Figure 4b, d). At equilibrium, under the most stressful conditions (red lines in Figure 4b, d), synergistic interactions occurred in the long‐term, while under lower stressor intensities we observed additive or antagonistic interactions in the long term (e.g., yellow and light orange lines in Figure 4b, d).

Population growth trajectories (and, thus, interaction type and strength) also depended on initial conditions for both seagrass (biomass) and consumer (density) (Figure 5). For example, when both consumer density and seagrass biomass were low (Figure 5a, b – point 1), the strength of the interactions (Figure 5c) were lower compared with when the consumer density was high relative to seagrass biomass (Point 4), thus the trajectory of the interaction started at a different point. Additionally, interaction trajectories could approach the long‐term interaction state in a qualitatively different way depending on the initial conditions, even when they those initial conditions were similar. This is because the instantaneous trajectories of seagrass biomass and consumer density differed among the treatments, which could lead to a fast divergence in observed biomass across treatments (Figure 5a, b).

### Sensitivity analysis

Variation in parameter values could alter interaction type or strength (SI Figures 1–5), but our findings were robust to the numerous alternate model functional forms (SI Figures 6–9). The magnitude of stressor interactions varied between models, but the overall interaction type remained consistent for all model runs at maximum stressor levels. At intermediate and low stress, some interaction types changed – especially during transient phases (SI Figure 7) – but the magnitude of effect was small (SI Figures 6–9). This tendency for interaction type to fluctuate between synergistic and antagonistic was especially pronounced for the Holling Type II consumer‐resource model. Such models exhibit limit cycles for certain parameter sets, and we found that these limit cycles could induce persistent cycling between synergy and antagonism (SI Figure 10).

## DISCUSSION

Non‐additivity in stressor effects should no longer be surprising as synergies and antagonisms are common in both experiments and in nature (Mantyka‐Pringle et al., 2019; Murdoch et al., 2020). Our analyses show how, across multiple levels of biological organisation, the same ecological and physiological processes could underpin additive, synergistic, or antagonistic stressor interactions. The classification and strength of stressor interactions depended on when during the experiment the interaction was measured, the magnitude of stressor effects and initial conditions. For instance, at equilibrium and under low stressor magnitudes, antagonistic or additive interactions were more common but at high stressor magnitudes, synergies always occurred. Such context‐dependencies suggest that seeking generalities in stressor responses by classifying interactions will often be of limited value, and could lead to ineffective or counter‐productive management (Brown et al., 2013). This highlights the importance of a mechanistic understanding of the processes that lead to changes in the biological endpoint of interest (De Laender, 2018), particularly when experiments do not match the spatial and temporal scales of relevance for management (as they typically do not). Our study also offers a plausible explanation for why previous meta‐analyses of experimental results have struggled to identify predictors of non‐additive interactions in the natural environment (Burgess et al., 2021; Côté et al., 2016; Crain et al., 2008; Jackson et al., 2016), and why, when they do, those predictors are not consistent across meta‐analyses.

Interaction type varied depending on when during the model run the response was measured, highlighting the challenges that need to be overcome when designing experimental tests of multiple stressors. Short‐term responses to multiple stressors may not even qualitatively reflect their long‐run responses (Leuzinger et al., 2011). Yet, most experiments on multiple stressors are conducted over short timeframes where transient dynamics prevail, and conclusions may be sensitive to initial experimental conditions. For example, the median experimental duration was 3 days (range: 2–134 days) in a meta‐analysis of 112 studies across terrestrial, freshwater, and marine ecosystems (Darling & Côté, 2008). The median experiment duration was 57 days in recent work assessing multiple stressor effects on freshwater fish (Lange et al., 2018), whereas it was 30 days for seagrass (Ostrowski et al., 2021). Nevertheless, we acknowledge that shorter experimental durations are likely to be suitable for some response types (e.g. mortality vs. growth) and target organisms (e.g. those with faster generation times) and depending on the types of stressors being applied (e.g. pulse vs. press – Jackson et al., 2021).

Here, we used PBMs to consider different interaction types during transient phases before the system’s asymptotic state was reached. To facilitate interpretation of shifts in stressor interaction type, we focused on responses in deterministic systems with a fixed equilibrium (but see SI Figure 10). However, we recognize that ecological systems are rarely at equilibrium (Burton et al., 2020; Hastings et al., 2018) for several reasons: fluctuations in model parameters with environmental variability often imply that the theoretical equilibrium state is itself constantly fluctuating, and thus the system never settles to a fixed point; long‐run behaviour of complex systems may involve limit cycles or chaos rather than approach to an equilibrium (Coulson, 2021); and long‐run equilibrium conditions themselves may be subject to long‐term trends as a consequence of the increasing magnitudes of stressor interactions. For our consumer‐resource model, interactions could fluctuate from antagonistic to synergistic and back again, when the approach to equilibrium was oscillatory (SI Figure 3). This suggests that, for systems with persistent cycles or chaotic dynamics, stressor interactions may well fluctuate indefinitely from synergistic to antagonistic as relative abundances of interacting populations change, as we observed for the Holling Type II consumer‐resource model (SI Figure 10). Similarly, systems with stochastic fluctuations in parameter values, which appear approximately additive on average, may also fluctuate between different interaction types.

More broadly, process models provide important advantages over phenomenological models usually used to analyse data, particularly when such models are integrated with empirical data (Connolly et al., 2017). For example, the parameters in process models (see Table 1) can be estimated independently of the data collected in a multi‐stressor experiment, and thus be used to predict system dynamics, whereas phenomenological models typically can only describe dynamics after the fact. Indeed, because PBMs represent a hypothesized causal structure of a system, they can be used to predict system behaviour under novel conditions (e.g. novel combinations of stressors or stressor magnitudes – Côté et al., 2016). When used naively, however, incorrect underlying assumptions can lead to flawed conclusions (Pirotta et al., 2022). We acknowledge that our models made several assumptions (i.e. no photo‐inhibition at high irradiance) and omitted some processes that could be important in seagrass systems. Consequently, model results may not reflect realistic multiple stressor impacts for seagrass systems in nature. However, this highlights the importance of confronting the predictions of process models with empirical data, because when such predictions fail, they imply that the hypothesized mechanistic structure of the system that the model represents is inadequate. Such insights can inform the formulation of new models, and thereby enhance the system understanding.

Our results also support calls for more experimental evidence to characterise the functional relationships between stressors and response variables (Pirotta et al., 2022; Schäfer & Piggott, 2018). This requires experiments with treatments across gradients of stress – rather than in just two levels – to more accurately identify the processes that underpin how stressors interact (Jackson et al., 2021; Pirotta et al., 2022) and to predict the ecological outcomes of multiple stressors in natural ecosystems (also see Galic et al., 2018). Currently, most multiple stressor studies apply stressors at only two levels (excluding controls; Ostrowski et al., 2021). Our findings highlight the need to move away from short‐term experiments testing very few levels of stressors, and echo recent theoretical work suggesting that care must be taken when attributing interaction types because the combination of stressor, model parameters impacted and stressor magnitude can affect the interaction type (Haller‐Bull & Bode, 2019). Although our model makes several assumptions (SI Table 1) that could alter the magnitude of change in our response variables under natural conditions, the key finding – that interaction type varies with timeframes and stressor magnitudes – remains a clear conclusion.

Our inclusion of a consumer‐resource model supports recent calls for research examining interactions at higher levels of biological organisation (Orr et al., 2020), supporting previous work demonstrating that species interactions can change stressor interaction types (Beauchesne et al., 2021; Thompson et al., 2018). Indeed, species interactions can amplify or dampen the sensitivity of natural systems to stressors. For instance, in seagrass ecosystems consumer presence mitigated seagrass losses from temperature stress and high nutrients by reducing epibiont biomass (Brodeur et al., 2015). Species interactions add further complexity when assessing multiple stressor effects and must be considered in future work. The inclusion of such species interactions in experiments, models and theoretical frameworks will lead to a more accurate and holistic understanding of stressor interactions and realistic impacts to ecosystems, although we acknowledge the logistical and financial constraints associated with conducting such experimental studies.

We found strong evidence that the same stressor interaction can vary from antagonistic, to additive, to synergistic depending on the response variables examined and the time frame over which responses are measured. We find some evidence that under higher stress, interactions may tend more towards synergy as systems approach equilibrium. Our findings highlight the need to understand how context affects the impact of multiple stressors more broadly, for example, due to the temporal dynamics of stressors (Jackson et al., 2021). Ecosystem conservation and effective management of multiple stressors requires greater integration of process modelling, laboratory studies and field data. Calibrating PBMs and then generating predictions that can be tested against data under controlled laboratory or field conditions can yield richer insights than either approach alone, as discrepancies can identify gaps in our understanding of the causal relationships among stressors and responses. Field experiments can further tell us how well the mathematical models approximate the dynamics of natural systems or if there may be additional factors whose incorporation may be required to draw inferences that are actionable in real‐world conservation and management contexts (Burd & Dunton, 2001). For continued progress in multiple stressor research, it is imperative to better leverage process‐based approaches and to maximize what we can learn from them by more comprehensively integrating them with experimental research.

## AUTHOR CONTRIBUTION

MPT, RA, MDC, RC, SC, IMC, FDL, MJ, MK, CMP, RBS, MS, PVDB, CJB, CJB, IMC and RC conceived of this study. The study was designed during a collaborative workshop attended by MPT, RA, SC, FDL, MJ, MK, CMP, RBS and PVDB. MPT, CJB and MDC performed modelling experiments with contributions from SRC, FDL and RBS. MPT and CJB wrote the first draft of the manuscript, and all authors contributed substantially to revisions.

### PEER REVIEW

The peer review history for this article is available at https://publons.com/publon/10.1111/ele.14013.

## Supporting information

Supplementary Material

## Data Availability

No new data were used in this analysis. All data used in analyses are available in the manuscript. The code used in this article is on Zenodo (https://doi.org/10.5281/zenodo.6419933).
