# Supplementary material for: Interactive effects of multiple stressors vary with consumer interactions, stressor dynamics and magnitude
Source: Ecol Lett. 2022 Apr 27;25(6):1483–96. doi: 10.1111/ele.14013 (PMC9320941; doi:10.1111/ele.14013)
Supplement: Supplementary file 1 — Supplementary Material [file ELE-25-1483-s001.docx]

**Supplementary Information**

Interactive effects of multiple stressors vary with consumer interactions, stressor dynamics and magnitude

**Authors:** Turschwell MP^1*^, Connolly SR^2,3^, Schäfer RB^4^, De Laender F^5^, Campbell MD^1^, Mantyka-Pringle C^6,7^, Jackson M^8^, Kattwinkel M^4^, Sievers M^1^, Ashauer R^9, 10^, Côté IM^11^, Connolly R^1^, van den Brink PJ^12,13^ & Brown CJ^1^.

^1^ Coastal and Marine Research Centre, Australian Rivers Institute, School of Environment and Science, Griffith University, Gold Coast, Qld, Australia

^2^ Naos Marine Laboratories, Smithsonian Tropical Research Institute, Balboa Ancón, Republic of Panama

^3^ College of Science and Engineering, James Cook University, Townsville, Australia

^4^ Quantitative Landscape Ecology, iES—Institute for Environmental Sciences, University Koblenz-Landau, Landau in der Pfalz, Germany

^5^ Research Unit of Environmental and Evolutionary Biology, Namur Institute of Complex Systems, and Institute of Life, Earth, and the Environment, University of Namur, Rue de Bruxelles 61, 5000 Namur, Belgium

^6^ Wildlife Conservation Society Canada, Whitehorse, Yukon Territory, Canada

^7^ School of Environment and Sustainability, University of Saskatchewan, Saskatoon, Saskatchewan, Canada

^8^ Department of Zoology, University of Oxford, Oxford, UK

^9^ Environment Department, University of York, Heslington, York YO10 5DD, UK

^10^ Syngenta Crop Protection AG, Basel, 4002, Switzerland

^11^ Earth to Ocean Research Group, Department of Biological Sciences, Simon Fraser University, Burnaby, BC V5A 1S6, Canada

^12^ Aquatic Ecology and Water Quality Management Group, Wageningen University, PO Box 47, 6700 AA Wageningen, The Netherlands

^13^ Wageningen Environmental Research, PO Box 47, 6700 AA Wageningen, The Netherlands

**
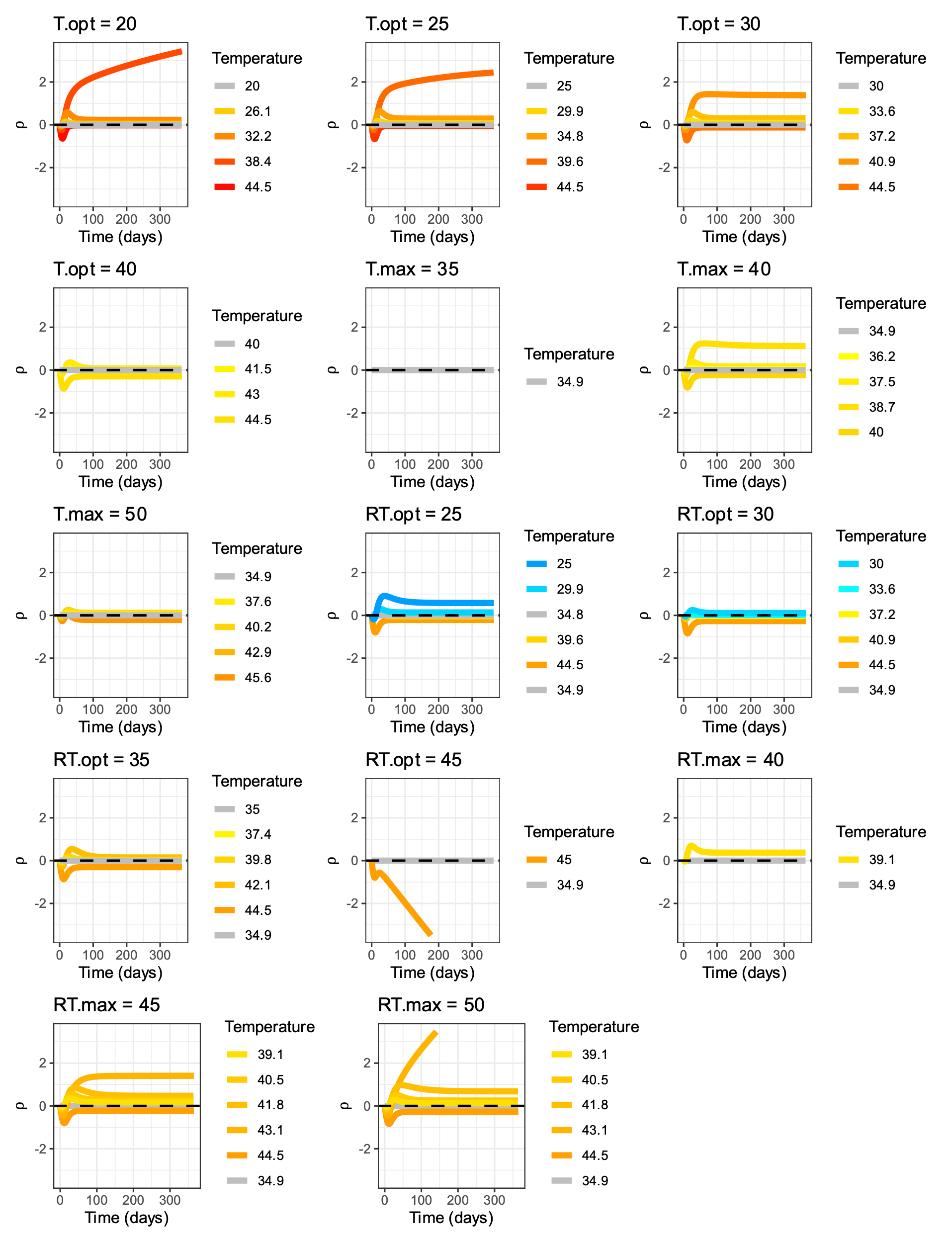
**

**SI Figure 1.** Sensitivity of the population sub model to changes in temperature for four input parameters, Topt, Tmax, RTopt, RTmax. Each line in the plots represents a changing temperature, where the colour scheme represents the difference between the temperature and Topt, where yellow and red represent cases where T > Topt, cyan and blue represent cases where T< Topt, and grey T ~= Topt (the colours are magnitude difference in degrees). is the interaction metric, where positive values indicate synergistic interactions between stressors, and negative values indicate antagonistic interactions. Dashed line at zero denotes where interactions are additive.


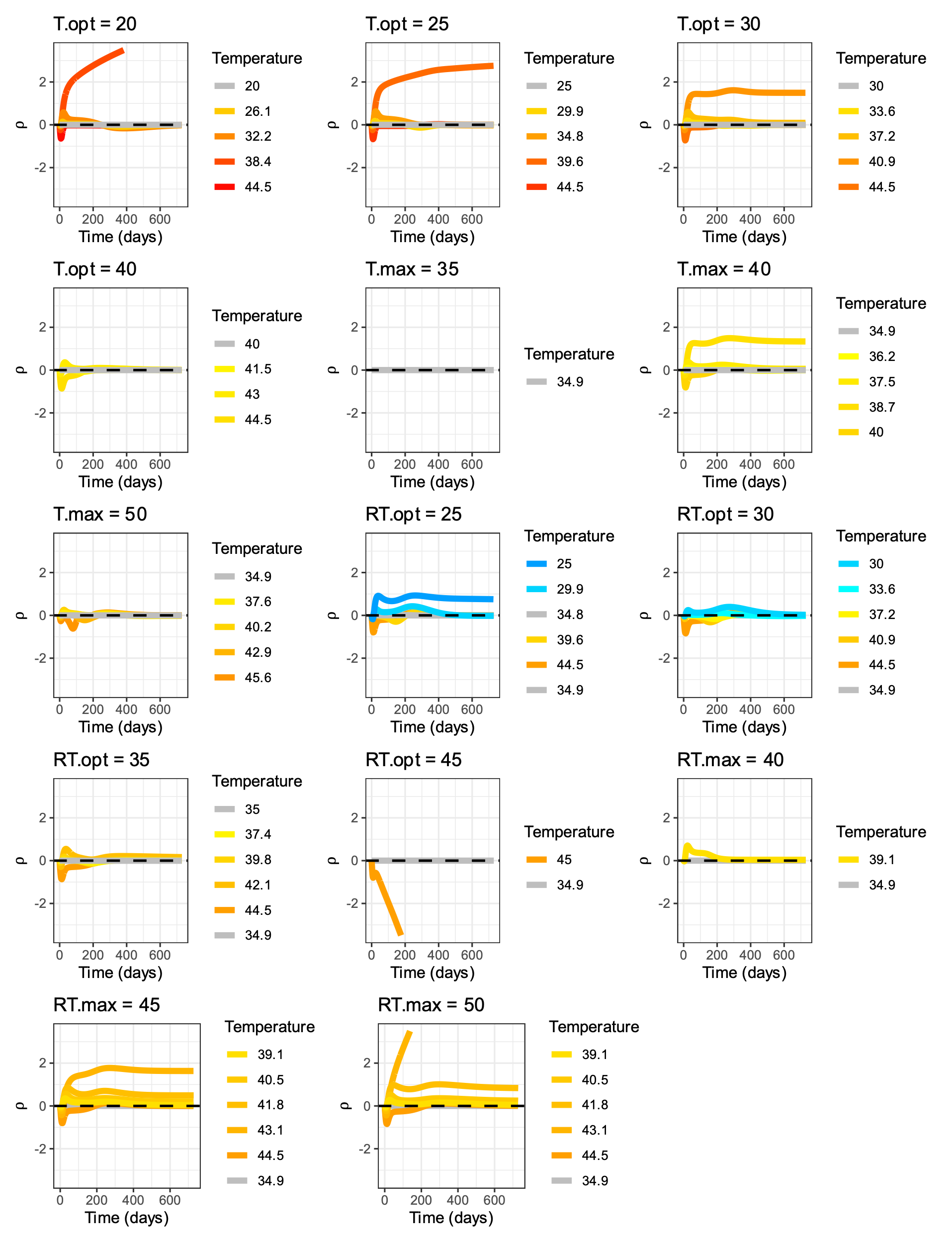


**SI Figure 2.** Sensitivity of the consumer-resource model to changes in temperature for four input parameters, Topt, Tmax, RTopt, RTmax. Each line in the plots represents a changing temperature, where the colour scheme represents the difference between the temperature and Topt, where yellow and red represent cases where T > Topt, cyan and blue represent cases where T< Topt, and grey T ~= Topt (the colours are magnitude difference in degrees). is the interaction metric, where positive values indicate synergistic interactions between stressors, and negative values indicate antagonistic interactions. Dashed line at zero denotes where interactions are additive.


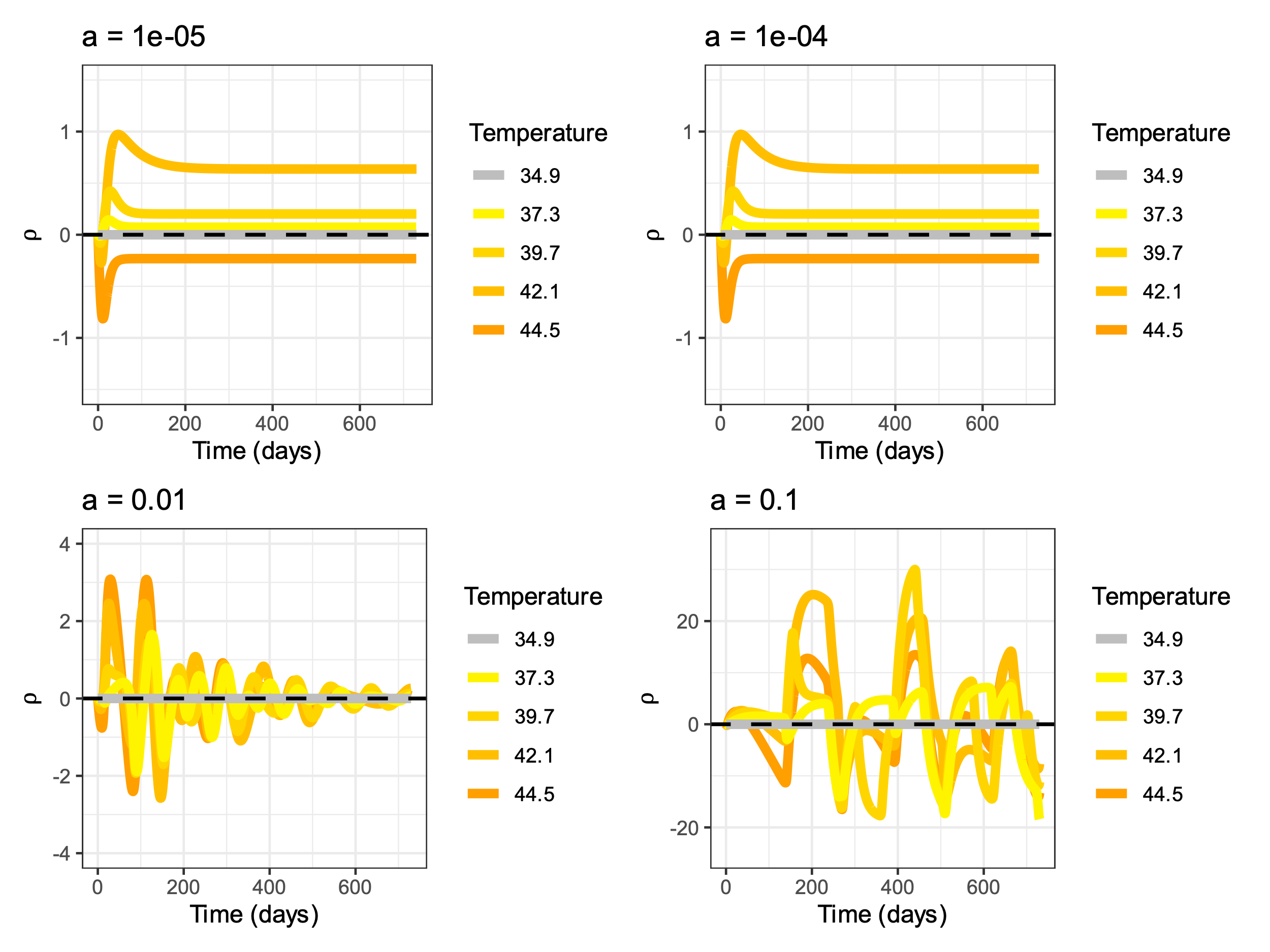


**SI Figure 3.** Sensitivity of the consumer-resource model to changes in attack rate. Each line in the plots represents a changing temperature, where the colour scheme represents the difference between the temperature and Topt, where yellow and red represent cases where T > Topt grey T ~= Topt (the colours are magnitude difference in degrees). is the interaction metric, where positive values indicate synergistic interactions between stressors, and negative values indicate antagonistic interactions. Dashed line at zero denotes where interactions are additive.


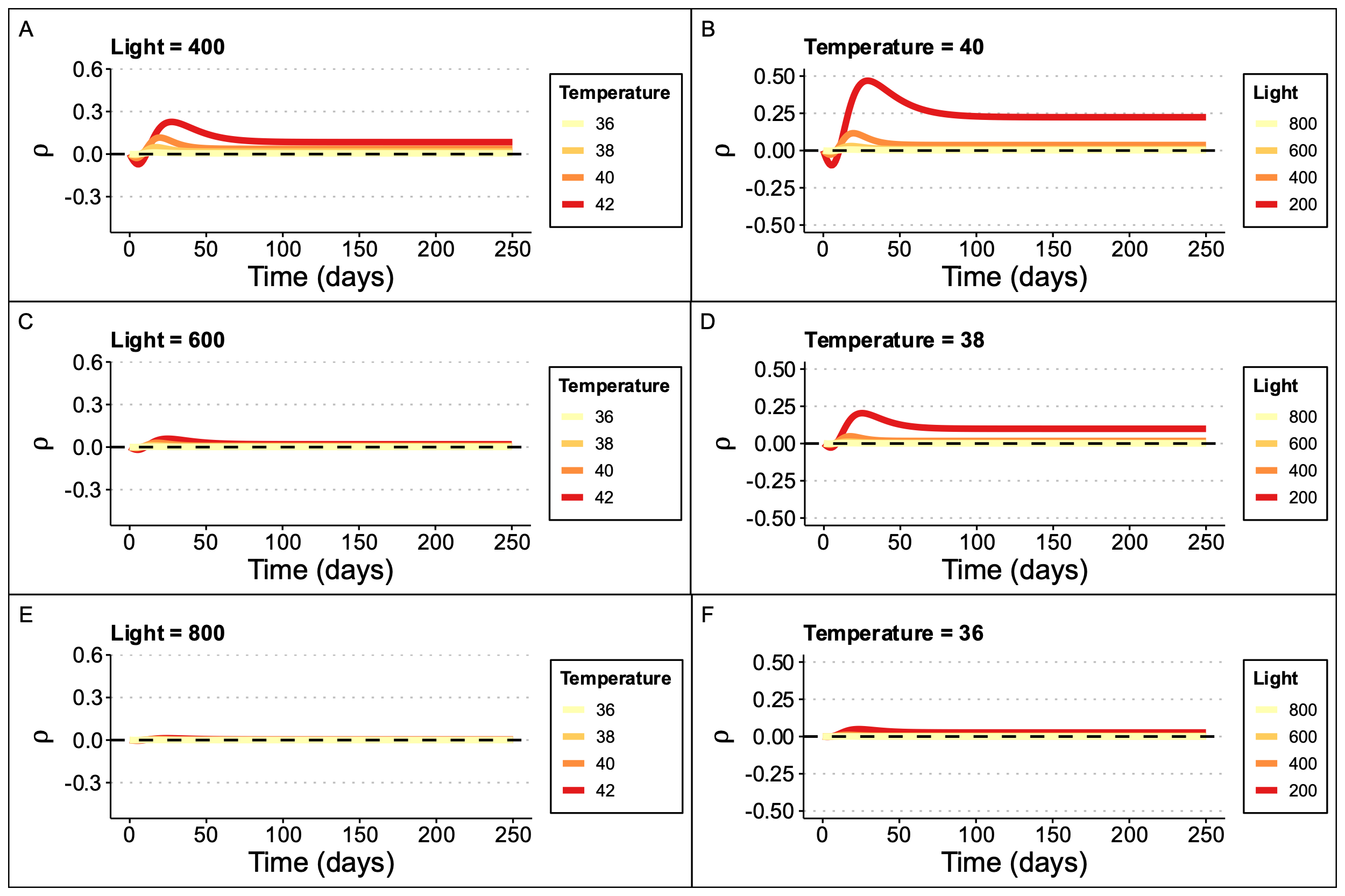


**SI Figure 4**. Sensitivity analysis highlighting combinations of fixed light with varying temperature (A, C, E), and fixed temperature with varying light (B, D ,F) stress tested for the population sub-model. is the interaction metric, where positive values indicate synergistic interactions between stressors, and negative values indicate antagonistic interactions. Dashed line at zero denotes where interactions are additive.


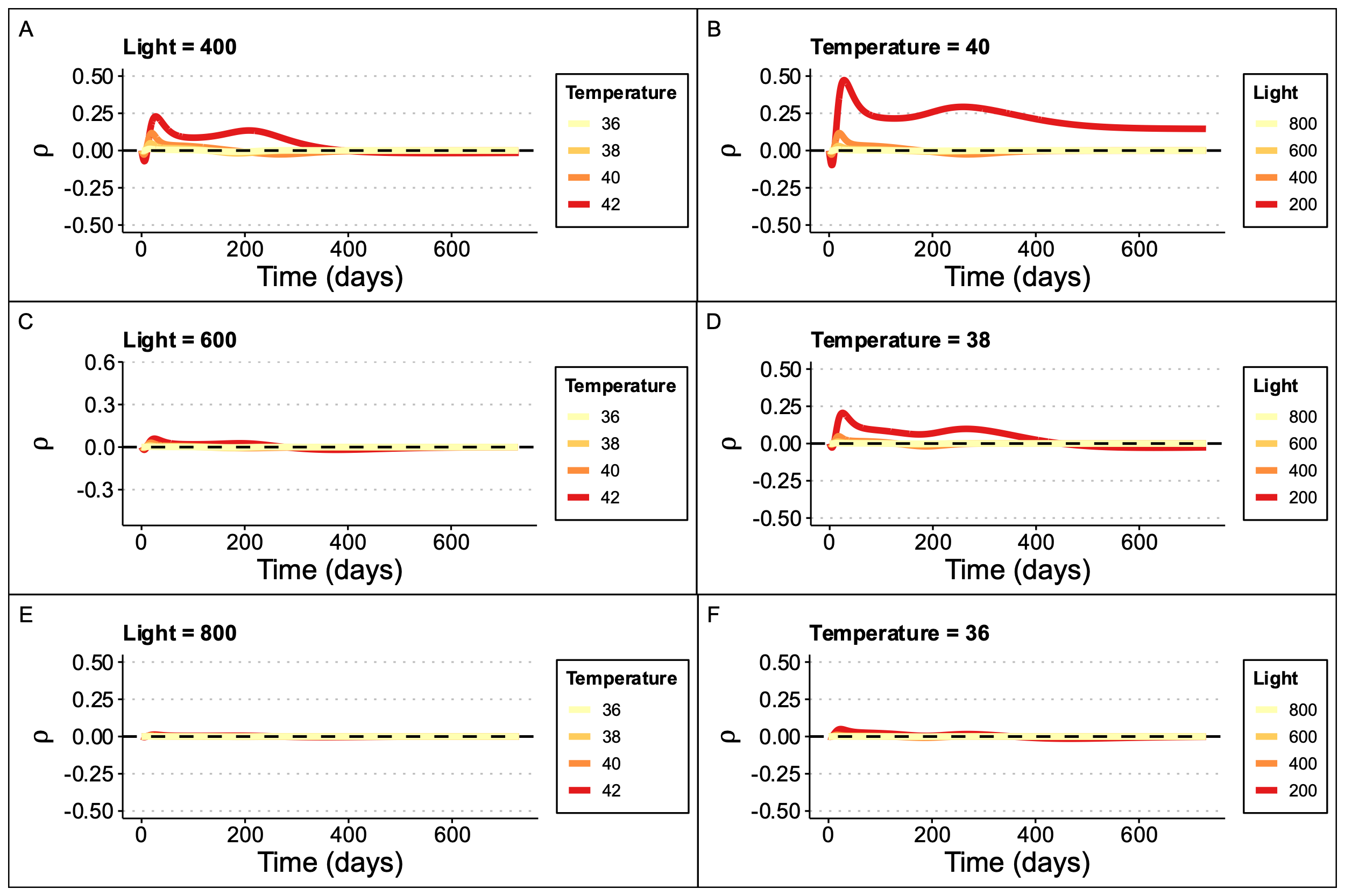


**SI Figure 5**. Sensitivity analysis highlighting combinations of fixed light with varying temperature (A, C, E), and fixed temperature with varying light (B, D ,F) stress tested for the consumer-resource model. is the interaction metric, where positive values indicate synergistic interactions between stressors, and negative values indicate antagonistic interactions. Dashed line at zero denotes where interactions are additive.


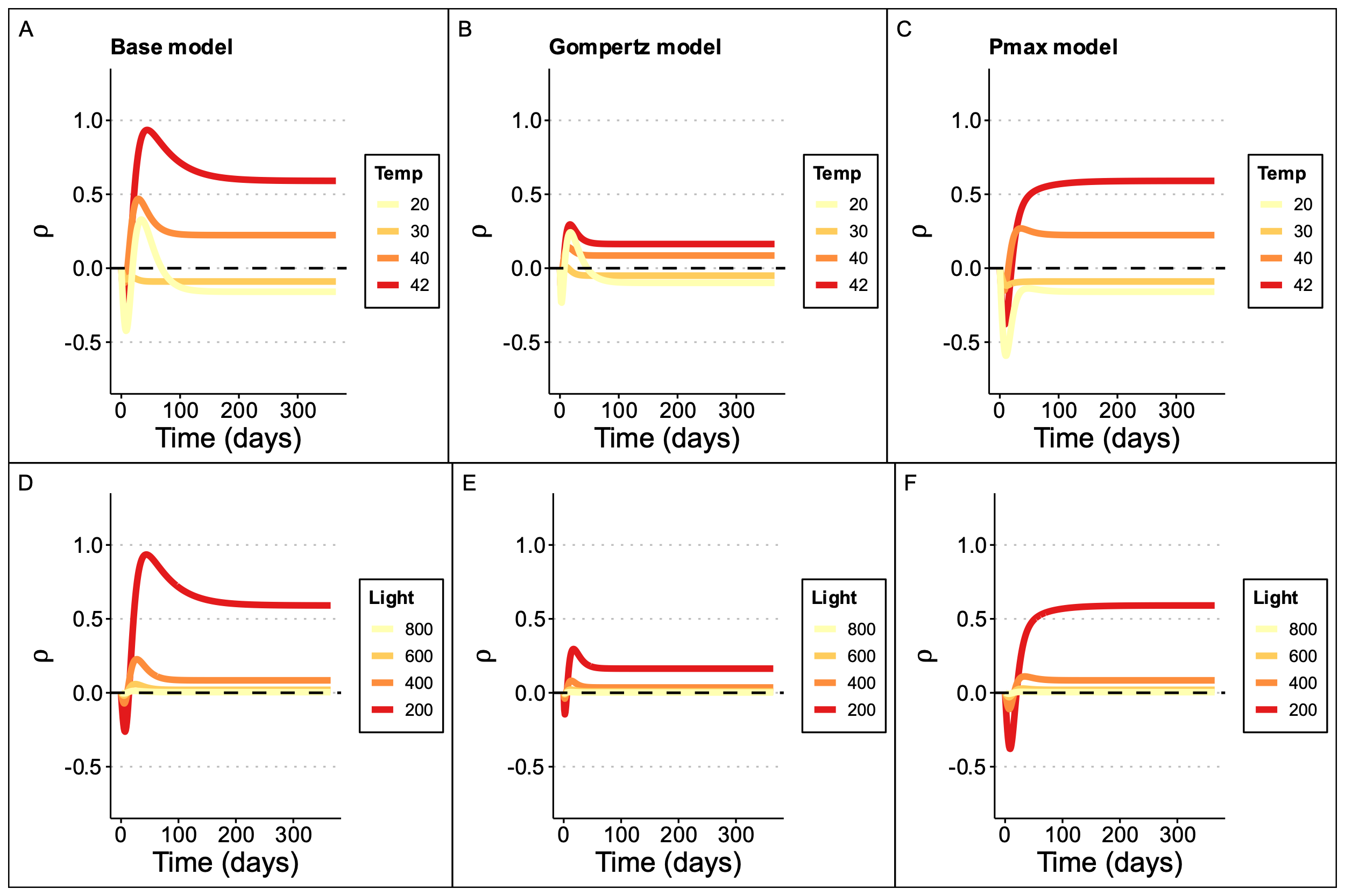


**SI Figure 6**. Population model sensitivity analysis highlighting different model functional forms for the base model (A, D), Gompertz model (B, E) and Pmax model (C, F). Stressor combinations are fixed light with varying temperature (A, B, C), and fixed temperature with varying light (D, E, F). is the interaction metric, where positive values indicate synergistic interactions between stressors, and negative values indicate antagonistic interactions. Dashed line at zero denotes where interactions are additive.


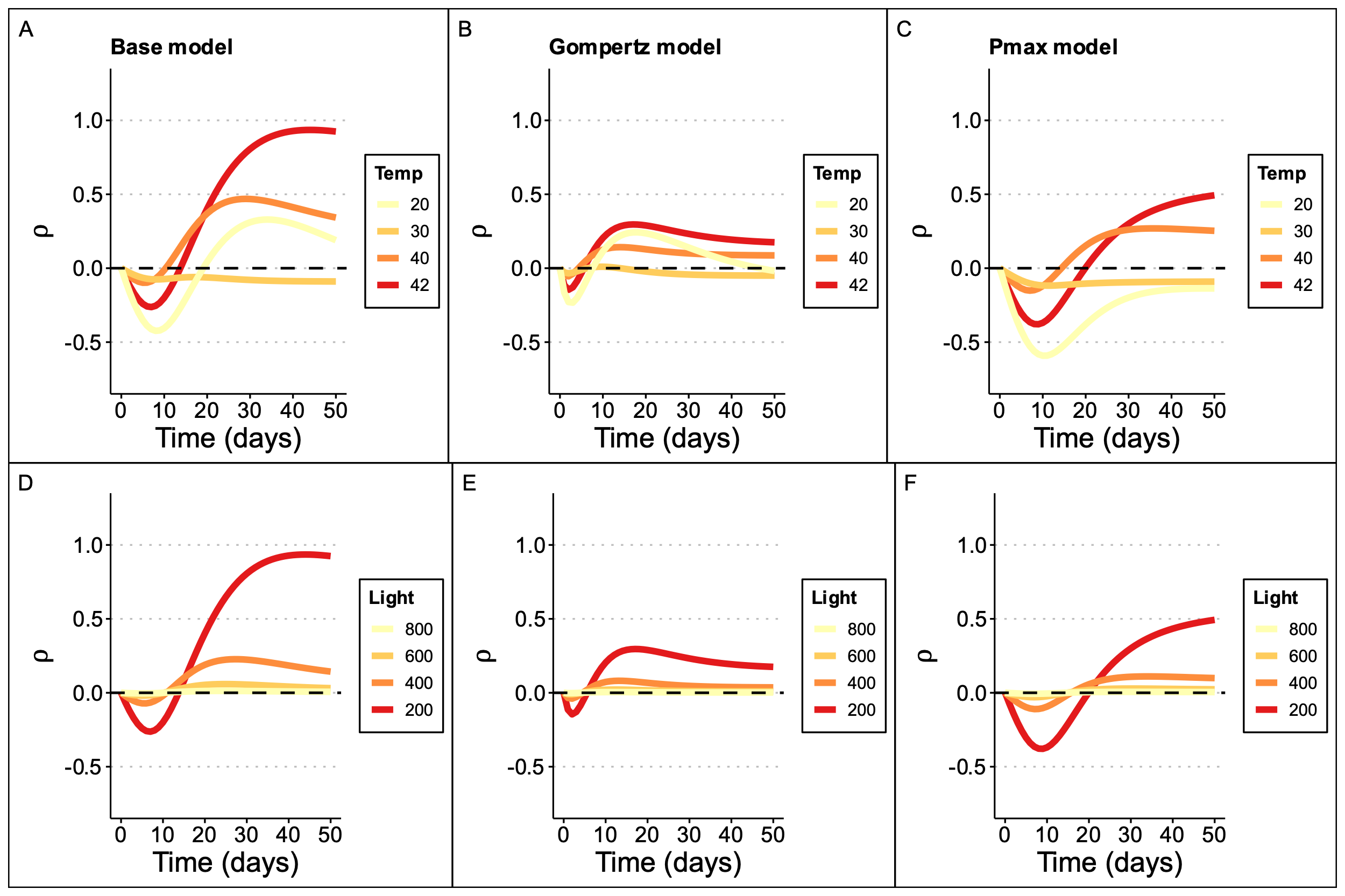


**SI Figure 7**. Population model sensitivity analysis highlighting different model functional forms for the base model (A, D), Gompertz model (B, E) and Pmax model (C, F) during transient phases (days 0-50). Stressor combinations are fixed light with varying temperature (A, B, C), and fixed temperature with varying light (D, E, F). is the interaction metric, where positive values indicate synergistic interactions between stressors, and negative values indicate antagonistic interactions. Dashed line at zero denotes where interactions are additive.


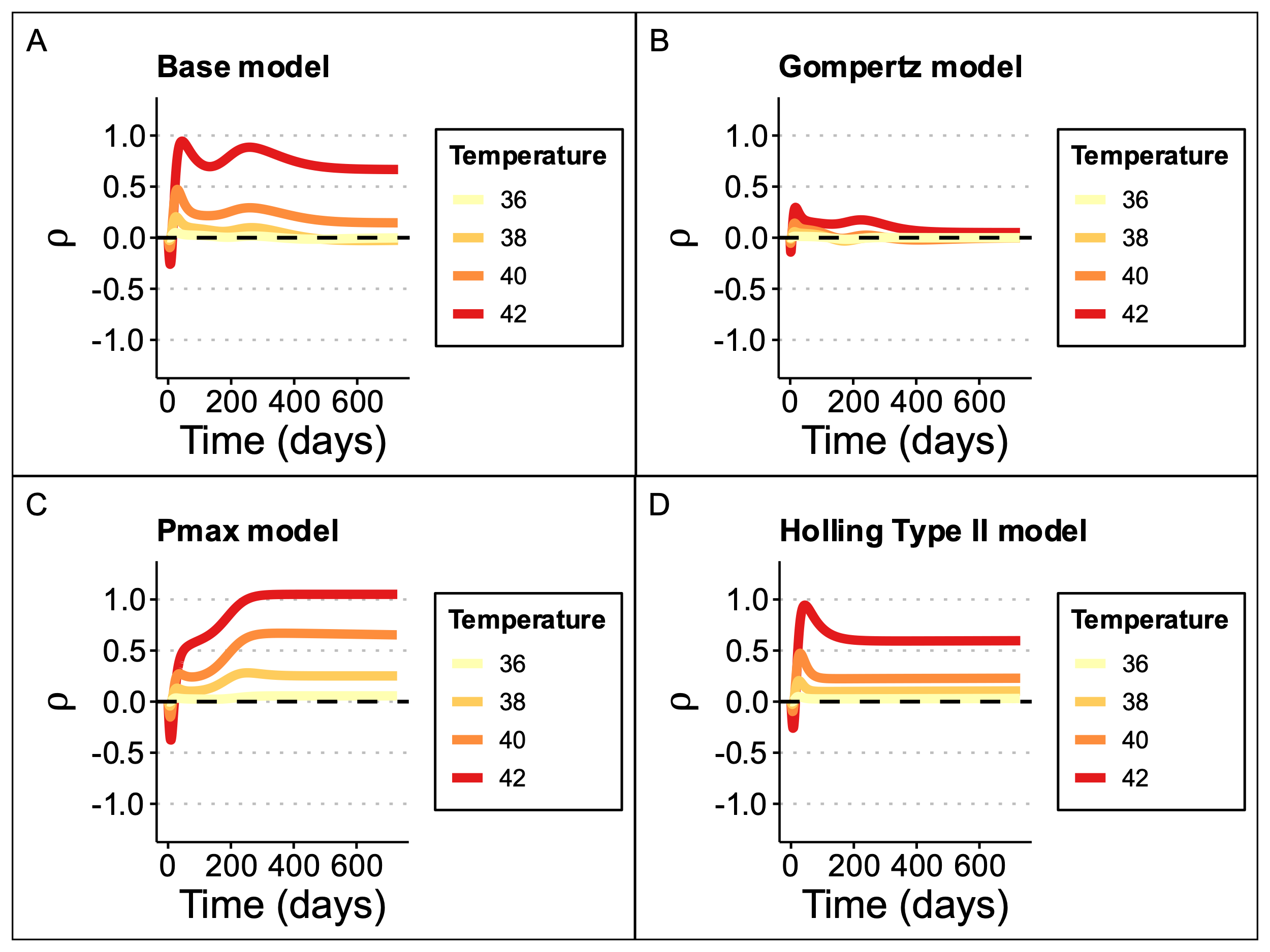


**SI Figure 8**. Consumer-resource model sensitivity analysis highlighting different model functional forms and the interactive effects of temperature and light. Stressor combinations are fixed light (200 *μ*mol) with varying temperature. is the interaction metric, where positive values indicate synergistic interactions between stressors, and negative values indicate antagonistic interactions. Dashed line at zero denotes where interactions are additive.


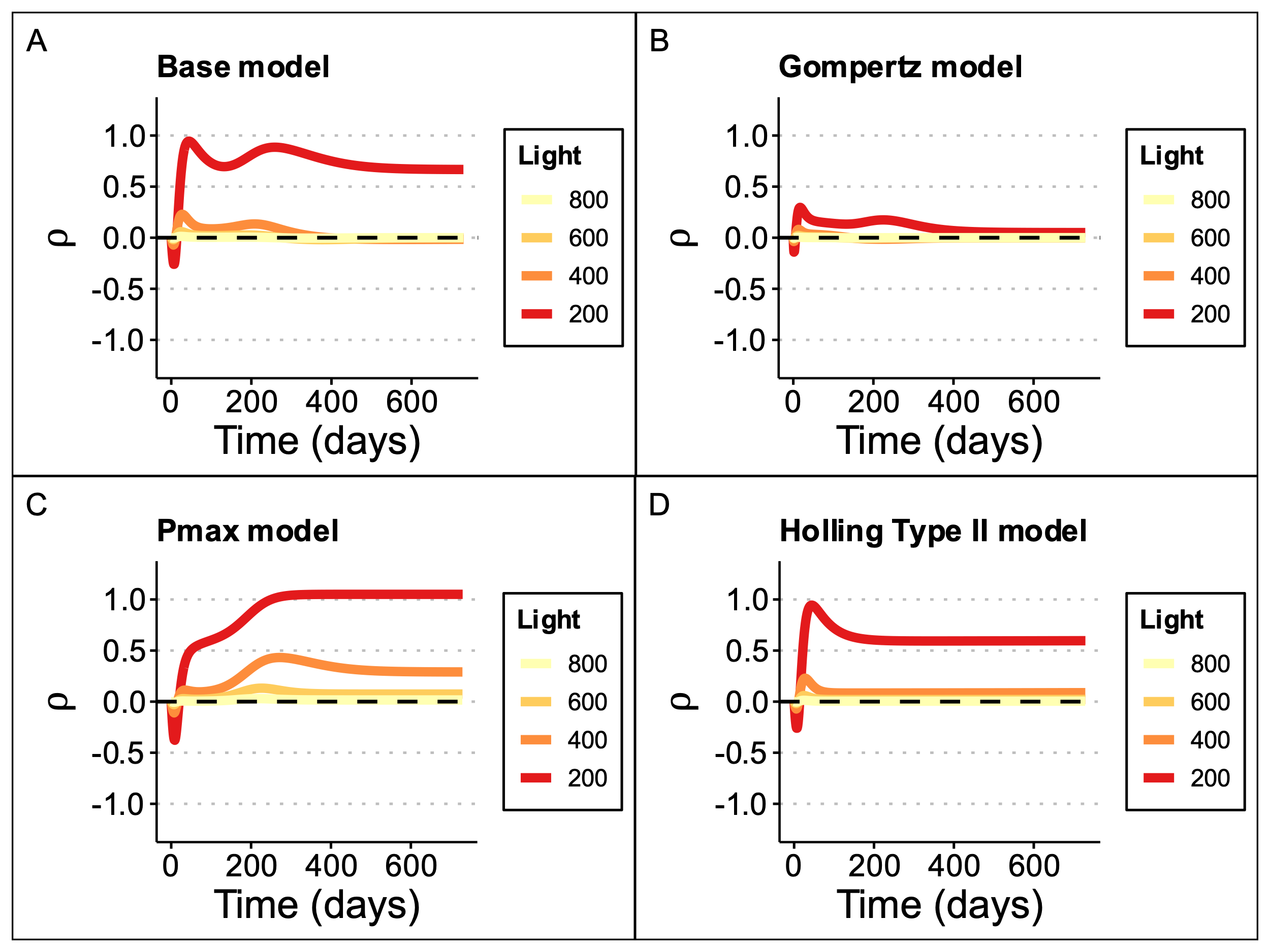


**SI Figure 9**. Consumer-resource model sensitivity analysis highlighting different model functional forms and the interactive effects of temperature and light. Stressor combinations are fixed temperature (42 °C) with varying light. is the interaction metric, where positive values indicate synergistic interactions between stressors, and negative values indicate antagonistic interactions. Dashed line at zero denotes where interactions are additive.


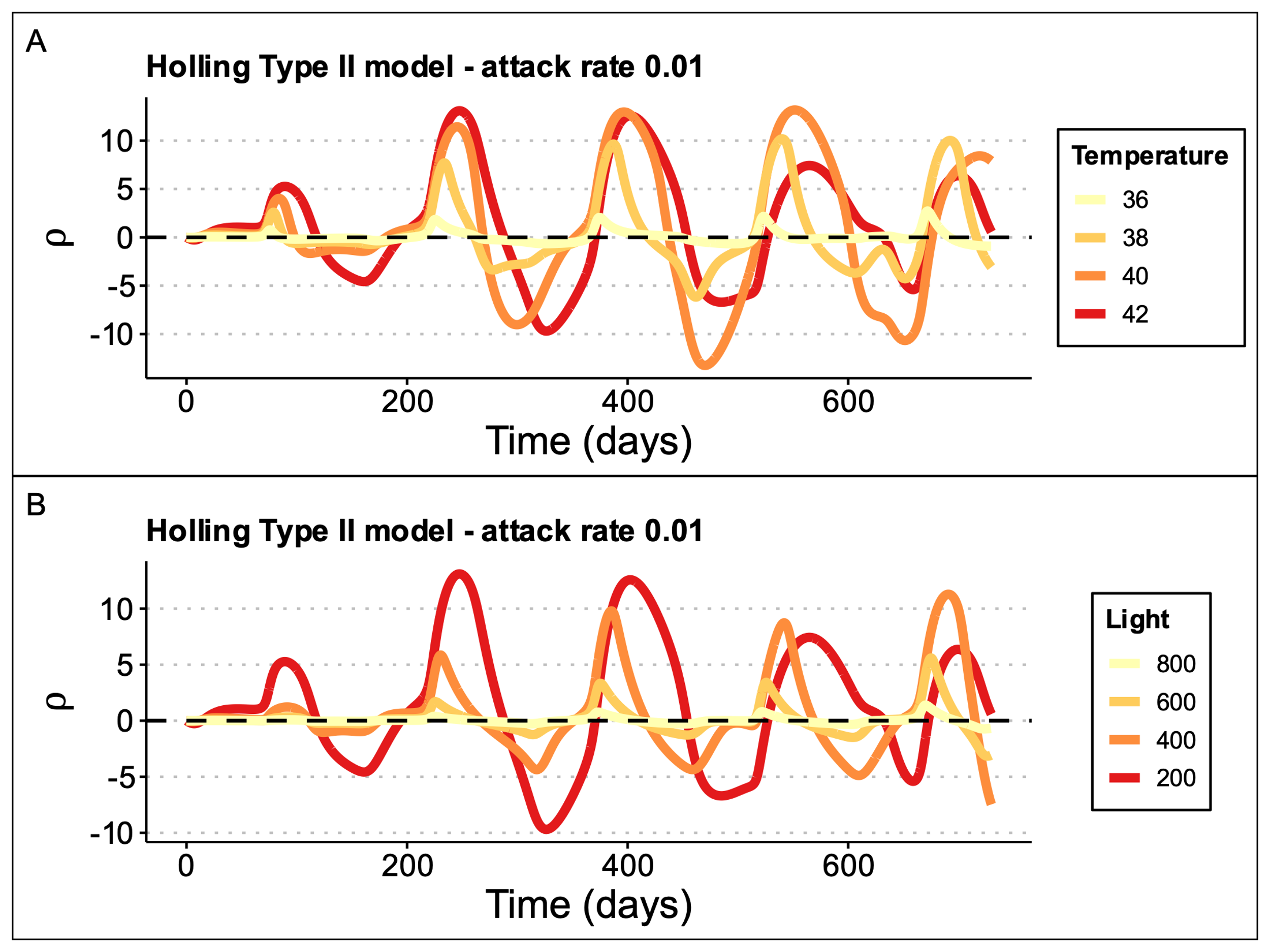


**SI Figure 10.** Sensitivity of the Holling Type II consumer-resource model to changes in attack rate, highlighting that interactions could fluctuate from antagonistic to synergistic and back again, when the approach to equilibrium was oscillatory. Stressor combinations are fixed light with varying temperature (A), and fixed temperature with varying light (B). is the interaction metric, where positive values indicate synergistic interactions between stressors, and negative values indicate antagonistic interactions. Dashed line at zero denotes where interactions are additive.

**SI Table 1.** Caveats and details to the modelling approach used.

| Caveat | Details |
| --- | --- |
| Seagrass biomass | Our model ignores below-ground biomass when modelling seagrass biomass (Vonk et al. 2015, Adams et al. 2018), and ignores interspecific competition. |
| Photosynthesis-light relationship | Our model also assumes a multiplicative relationship between photosynthesis being a function of light and temperature instead of Liebig’s law of the minimum. |
| Interaction metric | Our metric to classify interactions does not account for variability in the strength of the stressor interaction, meaning that any deviation from zero is classified as antagonism or synergy and that additive interactions may be less common than non-additive interactions. |
| Stressor Application | We applied static stressors and only allowed the response to vary through time, even though stressor intensities can change through time and space (Jackson et al. 2021), |

**Model stability**

We assessed model stability for both the population sub-model and the consumer-resource model to better understand the underlying dynamics of these models. These dynamics also give insights into the mechanisms that cause stressor interaction to change over time. We used analytical methods to determine the behaviour of the population sub-model. We found the system has two equilibrium points: $B^{*}=0$ and $B^{*}=\left( 1-\frac{R-m}{P_{max}} \right)B_{max}$, only one of which is stable. If $P_{max}-R-m<0$, the biomass *B* will approach zero, while if $P_{max}-R-m>0$ the biomass *B* will approach $B^{*}=\left( 1-\frac{R-M}{P_{max}} \right)B_{max}$.

We determined the behaviour of the consumer-resource model using a combination of analytical methods and a phase plane analysis using the *phaseR* package (Grayling 2014). This revealed the system would have one of three outcomes, which all lead to different final states: outcome 1, when $R+m+P_{max}<2$ and $\left( 1-R-m \right)B_{max}<\frac{vP_{max}}{ac}$, *X* goes extinct and it becomes the population-sub model; outcome 2, when $P_{max}<R+m$, *X* and *B* go extinct; or outcome 3, when $1>R+m+\frac{{vP}_{max}}{ac}$ and $1+\frac{v}{ac}<\frac{2v}{acB_{max}}+\frac{1}{P_{max}}$, *X* and *B* coexist and they approach a coexistence equilibrium point at $\left( \frac{v}{ac},\frac{\left( 1-R-m-\frac{{vP}_{max}}{ac} \right)}{aS} \right)$. The outcome attained is based solely on the parameter set when the initial conditions are not an equilibrium.

**Finding the nullclines and the equilibrium points**

First, we solve for the equilibrium points by setting $\frac{dB}{dt}=\frac{dX}{dt}=0$.

$$\frac{dB}{dt}=0=P_{max} \left( 1-\frac{B}{B_{max}} \right)B-R\left( T \right)B-MB$$

$$\Rightarrow0= \frac{-P_{max}}{B_{max}}\left( B-\frac{1 -R-M-aSX}{P_{max}}B_{max} \right)B$$

Which gives B-nullclines $B=0$ and $B=\frac{1 -R-M-aSX}{\phi}B_{max}$. Rearranging the second of these gives that $X=\frac{1-R-M}{aS}-\frac{P_{max}B}{aSB_{max}}$

$$\frac{dX}{dt}=0=(acSB-\nu S)X$$

Which is X-nullclines at $B= \frac{\nu}{ac}$ and $X=0$. By looking at where the B-nullclines intersect with the X-nullclines, we get three equilibrium points $(B^{*},X^{*})$ at $\left( 0,0 \right), \left( \frac{B_{max}(1-R-M)}{P_{max}}, 0 \right)$ and $\left( \frac{\nu}{ac}, \frac{\left( 1-R-M-\frac{P_{max}\nu}{ac} \right)}{aS} \right)$. There is no fourth equilibrium point because $B=0$ and $B= \frac{\nu}{ac}$ is impossible.

**Stability of equilibrium points**

Now we have that the Jacobian Matrix $J(B, X)$ of the system is

$$J\left( B, X \right)=\left( \begin{matrix} -\frac{2\phi}{B_{max}}B+P_{max}-R-M-aSX & -aSB \\ acSX & acSB-\nu S \end{matrix} \right)$$

We use the Jacobian to find the stability of the equilibrium points. Firstly, we look at the stability of $(B^{*},X^{*})=\left( 0,0 \right).$ Where,

$$J\left( 0, 0 \right)=\left( \begin{matrix} P_{max}-R-M & 0 \\ 0 & -\nu S \end{matrix} \right)$$

Which is a diagonal matrix so the eigenvalues can be read of the diagonal. This reveals this equilibrium is stable when $\phi<R+M$, because we always have $-\nu S<0$ (i.e., having food has a positive effect on the consumer). Secondly, we look at the stability of the equilibrium point $(B^{*},X^{*})=\left( \frac{B_{max}(1-R-M)}{P_{max}}, 0 \right) .$ Where,

$$J\left( \frac{B_{max}(1-R-M)}{P_{max}}, 0 \right)=\left( \begin{matrix} -\frac{2P_{max}}{B_{max}}\frac{B_{max}(1-R-M)}{P_{max}}+P_{max}-R-M & -aS\frac{B_{max}(1-R-M)}{P_{max}} \\ 0 & acS\frac{B_{max}(1-R-M)}{P_{max}}-\nu S \end{matrix} \right)$$

$$=\left( \begin{matrix} -2+2R+2M+P_{max}-R-M & -aS\frac{B_{max}(1-R-M)}{P_{max}} \\ 0 & acS\frac{B_{max}(1-R-M)}{P_{max}}-\nu S \end{matrix} \right)$$

$$=\left( \begin{matrix} -2+R+M+P_{max} & -aS\frac{B_{max}(1-R-M)}{P_{max}} \\ 0 & acS\frac{B_{max}(1-R-M)}{P_{max}}-\nu S \end{matrix} \right)$$

This is an upper triangular matrix so you can read the eigenvalues of the diagonal. So $(B^{*},X^{*})$ is stable when $R+M+P_{max}<2$ and $\left( 1-R-M \right)B_{max}<\frac{\nu P_{max}}{ac}$, otherwise it is unstable. Finally, we solve for the third equilibrium point $(B^{*},X^{*})=\left( \frac{\nu}{ac}, \frac{\left( 1-R-M-\frac{P_{max}\nu}{ac} \right)}{aS} \right)$. Where,

$$J\left( \frac{\nu}{ac}, \frac{\left( 1-R-M-\frac{P_{max}\nu}{ac} \right)}{aS} \right)=\left( \begin{matrix} -\frac{2P_{max}}{B_{max}}\frac{\nu}{ac}+P_{max}-R-M-aS\frac{\left( 1-R-M-\frac{P_{max}\nu}{ac} \right)}{aS} & -aS\frac{\nu}{ac} \\ acS\frac{\left( 1-R-M-\frac{P_{max}\nu}{ac} \right)}{aS} & acS\frac{\nu}{ac}-\nu S \end{matrix} \right)$$

$$=\left( \begin{matrix} -\frac{2P_{max}\nu}{acB_{max}}+P_{max}-\left( 1-\frac{P_{max}\nu}{ac} \right) & -S\frac{\nu}{c} \\ c\left( 1-R-M-\frac{P_{max}\nu}{ac} \right) & 0 \end{matrix} \right)$$

Now, for the $(B^{*},X^{*})$ to be stable we need that $trace\left( J\left( (B^{*},X^{*}) \right) \right)<0$ and det($J\left( (B^{*},X^{*}) \right))>0.$ Which means $\left( B^{*},X^{*} \right)$stable, if and only if,

$$-\frac{2P_{max}\nu}{acB_{max}}+P_{max}-\left( 1-\frac{P_{max}\nu}{ac} \right)<0$$

$$\Rightarrow P_{max}+\frac{P_{max}\nu}{ac} <\frac{2P_{max}\nu}{acB_{max}}+1$$

$$\Rightarrow1+\frac{\nu}{ac} <\frac{2\nu}{acB_{max}}+\frac{1}{P_{max}}$$

and

$$c\left( 1-R-M-\frac{P_{max}\nu}{ac} \right)\left( S\frac{\nu}{c} \right)>0$$

$$\Rightarrow1-R-M-\frac{P_{max}\nu}{ac}>0$$

$$\Rightarrow1>R+M+\frac{P_{max}\nu}{ac}$$

If either of these conditions fails, $(B^{*},X^{*})$ is unstable.

**References**

Grayling, M. J. (2014). phaseR: An R Package for Phase Plane Analysis of Autonomous ODE Systems. R Journal, 6(2).
